# Sociodemographic and maternal predictors of elevated blood lead levels during pregnancy in Adjara, Georgia

**DOI:** 10.1097/EE9.0000000000000496

**Published:** 2026-06-16

**Authors:** Sophio Sikharulidze, Charlotta Rylander, William Michael Caudle, Rusudan Shavishvili, Nona Ephadze, Tinatin Gvelesiani, Tinatin Manjavidze

**Affiliations:** a School of Health Sciences, University of Georgia, Tbilisi, Georgia; b Department of Community Medicine, Faculty of Health Sciences, The Arctic University of Norway, Tromsø, Norway; c Department of Environmental and Occupational Health, Center for Neurodegenerative Disease, Rollins School of Public Health, Emory University, Atlanta, Georgia; d Adjara Public Health Center, Batumi, Georgia; e Tbilisi State Medical University, Tbilisi, Georgia.

**Keywords:** Pregnant women, Lead exposure, Blood lead levels, Maternal health, Adjara, Georgia

## Abstract

**Background:**

Exposure to lead is a major public health concern throughout the world, particularly for susceptible groups such as pregnant women and children. Despite recent declines in blood lead levels (BLLs), lead exposure remains a health issue in Georgia. The present study was designed to identify predictors of elevated BLLs in pregnant women in the Adjara region of Georgia.

**Methods:**

Data were collected from the state health program “Lead biomonitoring program of pregnant and breastfeeding women in Adjara” (2020–2023) and the Georgian Birth Registry. The study included 8203 women who gave birth to 8337 babies. BLL groups among pregnant women were defined as <5, 5–9.99, and ≥10 µg/dL. Differences in characteristics between BLL groups were assessed with a chi-square test for categorical data and analysis of variance for continuous data. Multivariable logistic regression was used for identifying predictors of elevated BLL.

**Results:**

In the study sample, 50.5% of the subjects had BLLs <5 µg/dL, 35.5% had BLLs 5–9.99 µg/dL, while 14.0% had BLLs ≥10 µg/dL. Women with primary school as their highest completed education (adjusted odds ratio [aOR] = 1.65, 95% confidence interval [CI] = 1.19, 2.30) were more likely to be associated with BLLs ≥5 µg/dL compared with those with higher education. Obesity versus normal weight (aOR = 1.32, 95% CI = 1.15, 1.51) was significantly associated with BLLs ≥5 µg/dL. Living in a rural area was associated with slightly higher odds of BLLs of ≥5 µg/dL (aOR = 1.25, 95% CI = 1.13, 1.29). BLL declined over the testing period.

**Conclusion:**

Elevated BLLs in pregnant women in Adjara remain an important public health issue. Low education, obesity, rural residency, and timing of BLL testing were significantly associated with BLLs ≥5 and ≥10 µg/dL. According to these findings, continued surveillance is required for interventions to reduce lead exposure in vulnerable populations.

**What this study adds**

This study provides the first comprehensive analysis of maternal blood lead levels (BLLs) during pregnancy in Adjara, Georgia, using merged data from the state lead surveillance program and the Georgian Birth Registry. We report the prevalence of elevated BLLs among pregnant women and identify sociodemographic and maternal predictors. BLLs were classified into three categories (<5, 5–9.99, and ≥10 µg/dL) and assessed using chi-square, analysis of variance, and multivariable logistic regression. Our findings highlight persistent exposure risks, reveal predictors of elevated BLLs, and identify opportunities to strengthen interventions for maternal and child health protection.

## Introduction

Lead, a well-known toxic metal, continues to be a widespread environmental contaminant, endangering human health globally.^1^ Although much has been achieved in the control and elimination of lead sources, such as the global phase-out of leaded petrol and the removal of lead in paint in many countries, the extensive history of use has resulted in sustained lead pollution, with measurable levels in humans.^2^ Pregnant women and fetuses are one of the groups with a high susceptibility to adverse health effects caused by lead exposure.^3^ Lead is a strong neurotoxin that can cross the placenta and compromise fetal development, increasing the risk of preterm birth, low birth weight, and gestational hypertension.^4^ Moreover, prenatal lead exposure can be secondary to the release of lead from the mother’s bones, resulting in higher exposure of the fetus in utero and consequently posing a greater health risk.^5^ The United States Center for Disease Control and Prevention designates BLL ≥3.5 µg/dL as the threshold for further clinical observation and management of pregnant women.^6^ While the global impetus for lead poisoning prevention seems to have lifted, lead poisoning prevention continues to be a focus in the United States and most European countries and remains a critical need in low- and middle-income countries, including Georgia.^7^

Georgia is situated at the dividing line between Europe and Asia and has a population of 3.75 million people.^8^ According to the 2018 Multiple Indicator Cluster Survey (MICS) conducted by the National Statistics Office of Georgia, in cooperation with UNICEF, and the National Center for Disease Control and Public Health, the prevalence of BLLs ≥5 µg/dL among children aged 2–7 years was 41.1% with the highest proportion in Adjara (85.4%), Guria (73.2%), and Samegrelo-Zemo Svaneti (71.2%).^9^ In 2020, the Adjara government launched a state health program to better understand the factors leading to elevated BLL among pregnant and lactating women. The current study aims to describe the demographic and maternal characteristics of women with elevated BLLs in Adjara from 2020 to 2023 and to identify predictors of elevated BLLs.

## Methods

### Lead biomonitoring program of pregnant and breastfeeding women in Adjara

Data on BLLs were provided by the lead biomonitoring program of pregnant and breastfeeding women in Adjara, administered by the Adjara Public Health Center in Batumi, Georgia. All pregnant and lactating women registered in Adjara, as Adjara residents, were eligible for BLL testing during pregnancy and breastfeeding. In 2020 and the first half of 2021, BLL testing was limited to women designated as high risk for lead exposure. However, after a year, the testing was expanded to include all pregnant women attending antenatal care (ANC). In our study, we included all women who had at least one BLL test during pregnancy, resided in Adjara and delivered during the study period.

### Georgian Birth Registry

The Government of Georgia initiated the implementation of the Georgian Birth Registry (GBR) in 2016, with technical assistance from the staff of the Arctic University of Norway and the support of UNICEF.^10^ The GBR is a completely electronic health system under the administration of the National Centre for Disease Control and Public Health, registering data for more than 99% of all Georgian births, as Georgian law mandates the registration of all pregnant women in the GBR. The GBR incorporates information on ANC attendance, obstetric history, and newborn health data. It includes more than 500 variables, including maternal sociodemographic characteristics and health conditions, as well as detailed information about fetal and neonatal outcomes. Every Georgian citizen is assigned a personal ID number, allowing the information to be linked within or across various health registries and databases.

### Ethical approvals

The present study was endorsed by the University of Georgia Bioethical Board (Reference number: 11-34422) and the Ethical Board of the National Center for Disease Control and Public Health (NCDC reference number: IRB#2023-002).

### Study sample

According to the data, 10,123 women identified through the lead biomonitoring program of pregnant and breastfeeding women between September 2020 and July 2023 (excluding duplicate entries, n = 7) were merged with GBR using a unique 11-digit personal identification number between January 1, 2020, and September 7, 2023.^11^ Two hundred ninety-two women were excluded because they had a BLL test but were not registered in the GBR, 27 women were excluded due to BLL testing before conception, 598 for testing after abortion or delivery, 914 for ongoing pregnancies at the time of testing, and 82 women whose pregnancies ended in abortion.

**Figure 1.**
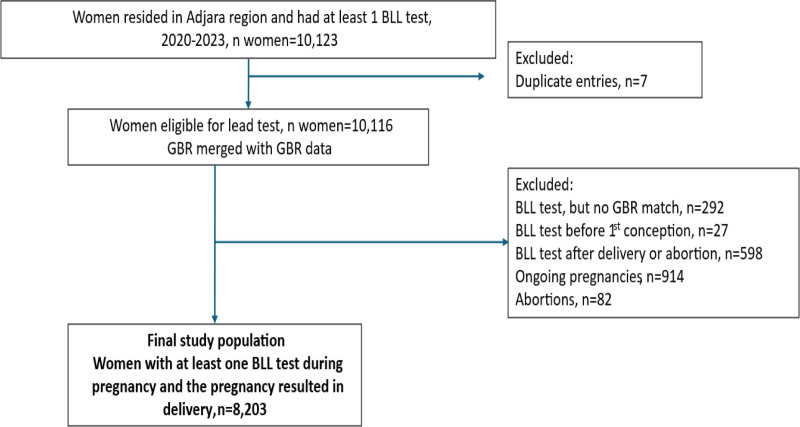
The flowchart of the study population. Data from the lead biomonitoring program of pregnant and breastfeeding women in Adjara and the Georgian Birth Registry, 2020–2023.

The final study sample included 8203 women, who had at least one BLL test during pregnancy, gave birth to 8337 newborns, and resided in the Adjara region during the study period (Fig. 1).

### Chemical analysis

Venous blood (6 mL) was drawn from pregnant women and collected in vacutainers containing EDTA buffer for trace element (lead) determination. Blood samples were sent to the Mediprime LLC Medical Laboratory in Tbilisi, Georgia, an ISO15189 certified medical laboratory.

For analysis, 100 μL of a whole blood sample was pipetted into an Eppendorf tube, and 100 μL of 2% ultra-pure nitric acid (HNO_3_, 70%, sublimed grade, ≥99.999% trace metals purity, MERCK, Darmstadt, Germany) and 900 μL of modifier solution were added. The mixture was vortexed at 5000 rpm for 1 minute to mix thoroughly. This was followed by transferring 300–500 μL of the final supernatant into another test tube that was subjected to atomic absorption spectrometry) analysis using a graphite furnace Agilent 280Z Atomic Absorption Spectrometer with Zeeman background correction (Agilent Technologies, Santa Clara, CA).

The modifier solution was prepared by mixing 400 mL of deionized water, 25 mL of 10% TRITONTM X-100 (v/v Alean Chemicals Co., Ltd., Taipei, Taiwan), 5 mL of 20% ultra-pure ammonium dihydrogen phosphate (NH_4_H_2_PO_4_) solution (99.9%, trace metals basis, Thermo Scientific, Waltham, Massachusetts), and 1 mL of concentrated HNO_3_ to make up to 500 mL with deionized water.

Blank blood samples (control, ACQ Science, Montreal, Canada) and certified reference material (Seronorm Trace Element Serum, Sero, Hvalstad, Norway) were included in the analysis to verify quality control of the measurements. The certified reference material concentration was invariably within ±10% of the certified value. The method detection limit for this method was 0.9 µg/dL.

### Variables

Variables collected for the analysis include BLL test results during pregnancy, trimester of pregnancy at the time of a BLL test, maternal age (categorized as ≤19, 20–29, 30–39, ≥40 years), highest completed education level at the time of childbirth (primary, secondary, higher), marital status (married, single, or unknown), body mass index (BMI) at the time of first antenatal visit (<18.5, 18.5–24.9, 25.0–29.9, or ≥30.0 kg/m^2^), residency (urban/rural), delivery type (cesarean section [CS]/vaginal), parity (primiparous/multiparous), and year of BLL test (2020, 2021, 2022, or 2023).

### Statistical analysis

Summary statistics (e.g., frequencies with percentages and means with standard deviations) were used to describe characteristics of the study sample.

Chi-square test was used to identify differences across BLL groups for categorical variables, including marital status, education, residency, parity, mode of delivery, and test year.

One-way analysis of variance was used to assess differences in maternal age between BLL groups. *P* value of <0.05 was considered statistically significant for each analysis.

To identify associations between maternal characteristics and elevated BLL, multivariable logistic regression analyses were conducted using binary outcomes: the first model BLL ≥5.0 µg/dL (yes/no) and the second model BLL ≥10.0 µg/dL (yes/no), as these are thresholds used in Georgian guidelines for defining elevated BLL. The adjusted models included all potential predictor variables, such as maternal age, education, marital status, BMI, urban residency, parity, and test year, as well as BLL as an outcome, the first model BLL ≥5.0 µg/dL and the second BLL≥10.0 µg/dL year. The statistical analyses were conducted with Stata software, version 18.

## Results

### Study sample characteristics

Of the included 8203 pregnant women residing in Adjara during the study period (2020–2023), 50.5% had BLLs <5 μg/dL, 35.5% had BLLs between 5 and 9.99 μg/dL, and 14.0% had BLLs ≥10 μg/dL. The mean maternal age was 29.4 years (standard deviation = 5.7). Women aged 20–29 years represented the majority of all BLL groups (54.7%), and the distribution of age groups did not differ significantly across BLL categories (*P* value = 0.3). In relation to education level, most women had completed secondary school at the time of childbirth. However, there were significantly fewer women with higher education in the group with BLLs ≥10 µg/dL than in the group with BLLs between 5 and 9.99 µg/dL (16.3% and 21.4%, respectively) and compared with the <5 µg/dL group (16.3% vs. 31.5%) (Table 1).

**Table 1.** Maternal and newborn characteristics of pregnant women by BLL groups in Adjara, 2020–2023

	BLL <5 µg/dL n (%)	BLL 5–9.99 µg/dL n (%)	BLL ≥10 µg/dL n (%)	*P* value	Total number of women
Mothers	4,145 (50.5)	2,910 (35.5)	1,148 (14.0)		8,203
Maternal age, mean (SD)				0.3	
≤19	126 (3.0)	91 (3.2)	31 (2.7)		248
20–29	2,216 (53.5)	1,638 (56.3)	633 (55.2)		4,487
30–39	1,655 (40.0)	1,096 (37.7)	449 (39.2)		3,200
≥40	148 (3.6)	85 (3.0)	35 (3.1)		268
Education				<0.001	
Primary	67 (1.7)	83 (2.9)	43 (3.8)		193
Secondary	2,156 (52.1)	1,900 (65.3)	829 (72.3)		4,885
Higher	1,303 (31.5)	622 (21.4)	187 (16.3)		2,112
Unknown	619 (14.9)	305 (10.5)	89(7.8)		1,013
Marital status				<0.001	
Married	2,445 (59.0)	1,543 (53.0)	598(52.1)		4,586 (55.9)
Single	110 (2.6)	80 (2.8)	34(3.0)		224 (2.7)
Unknown	1,588 (38.3)	1,287 (44.2)	516(44.9)		3,391 (41.4)
BMI at first visit, kg/ m^2^				<0.001	
<18.5	246 (6.0)	147 (5.1)	44 (3.9)		437 (5.5)
18.5–24.9	2,234 (53.9)	1,514 (52.1)	533 (46.4)		4,281 (52.2)
25.0–29.9	1,041 (25.1)	757 (26.0)	323 (28.2)		2,121 (26.0)
≥30.0	604 (15.0)	481 (16.7)	246 (21.5)		1,331 (16.3)
Urban residency				0.4	
Urban	2,840 (68.6)	1,954 (67.2)	773 (67.4)		5,567
Rural	1,305 (31.5)	956 (32.9)	375 (32.7)		2,636
Parity				0.01	
Primiparous	1,636 (39.5)	1,090 (37.5)	400 (35.0)		3,126
Multiparous	2,509 (60.1)	1,820 (63.0)	748 (65.2)		5,077
Delivery type				0.4	
CS (cesarean section)	2,442 (59.0)	1,742 (59.9)	1,743 (60.0)		5,927
Vaginal	1,703 (41.1)	1,168 (40.2)	1,169 (40.5)		5,209
Test year				<0.001	
2020	245 (6.0)	384 (13.2)	290 (25.3)		919
2021	1,674 (40.4)	1,687 (58.0)	693 (60.4)		4,054
2022	1,736 (42.0)	742 (25.5)	142 (12.4)		2,620
2023	490 (12.0)	97 (3.4)	23 (2.0)		610

In our cohort, 55.9% of women were married, and the proportion of married women was the highest for the BLL <5 µg/dL group (59.0%). The proportion of women with obesity (25.0–29.9 kg/m^2^) was significantly higher in the BLL ≥10 µg/dL group (21.5%) at their first ANC visit compared with women with BLL between 5 and 9.99 µg/dL (16.7%) and women with BLL<5.0 µg/dL (15.0%). Most women lived in an urban area (67%–68%), and there was no difference in urban residency across BLL groups. Multiparous women accounted for the majority in all three groups, with the lowest proportion in the BLL <5 µg/dL group (60.1%) and the highest in the BLL ≥10 µg/dL group (65.2%). The prevalence of CS was consistent across the BLL categories, with approximately 59%–60% of the women in each BLL category having given birth by CS. In all BLL categories, the highest proportion of women was screened in 2022 (40.4%–60.4%) (Table 1).

In the multivariate logistic regression analysis, we included the following variables: BLL (either ≥5.0 or ≥10.0 µg/dL), maternal age, education, marital status, BMI, urban residency, parity, and test year, as potential predictors of elevated BLL. Results from multivariable logistic regression suggest women with primary education had significant odds of having BLLs ≥5 µg/dL (adjusted odds ratio [aOR] = 1.65, 95% confidence interval [CI] = 1.19, 2.30) and BLLs ≥10 µg/dL (aOR = 1.66, 95% CI = 1.12, 2.47) when compared with women with higher education. Similarly, women with secondary education had increased odds for both BLL ≥5.0 µg/dL (odds ratio [OR] = 1.30, 95% CI = 1.16, 1.47) and BLL ≥10.0 µg/dL (OR = 1.39, 95% CI = 1.16, 1.66).

Compared with women with normal BMI (18.5–24.9 kg/m^2^), those with underweight (<18.5 kg/m^2^) had reduced odds of elevated BLLs, though not statistically significant for either BLL ≥5.0 µg/dL (OR = 0.87, 95% CI = 0.70, 1.07) or BLL ≥10.0 µg/dL (OR = 0.81, 95% CI = 0.58, 1.13). Women with overweight (25.0–29.9 kg/m^2^) had significantly higher odds of elevated BLLs (BLL ≥5.0 µg/dL: OR = 1.14, 95% CI = 1.02, 1.28; BLL ≥10.0 µg/dL: OR = 1.26, 95% CI = 1.08, 1.47), and the association was even stronger among women with obesity (BMI ≥30.0 kg/m^2^) (BLL ≥5.0 µg/dL: OR = 1.32, 95% CI = 1.15, 1.51; BLL ≥10.0 µg/dL: OR = 1.60, 95% CI = 1.34, 1.91).

Women living in rural areas had significantly higher odds of BLL ≥5.0 µg/dL compared with those in urban areas (OR = 1.25, 95% CI = 1.13, 1.29), while the association for BLL ≥10.0 µg/dL did not reach statistical significance (OR = 1.14, 95% CI = 0.99, 1.32).

There was no significant association between parity and BLL groups.

Compared with test year 2023, the odds of BLL ≥5.0 µg/dL were significantly higher in all previous years: 2020 (OR = 10.1, 95% CI = 7.82, 13.2), 2021 (OR = 5.2, 95% CI = 4.17, 6.45), and 2022 (OR = 1.98, 95% CI = 1.59, 2.47). The same trend was revealed for BLL ≥10.0 µg/dL, with the highest odds in 2020 (OR = 10.2, 95% CI = 6.52, 15.98), followed by 2021 (OR = 4.49, 95% CI = 2.91, 6.94) and 2022 (OR = 1.35, 95% CI = 0.86, 2.12), compared with 2023 (Table 2).

**Table 2.** Association between maternal characteristics and BLL groups, crude and adjusted odds ratio (OR) and 95% confidence intervals (CIs)

		BLL ≥5 (n = 4,058)			BLL ≥10 (n = 1,148)	
	n (%)	OR (95% CI)	aOR (95% CI)	n (%)	OR (95% CI)	aOR (95% CI)
Maternal age						
≤19	122 (3.0)	0.95 (0.73, 1.22)	0.85 (0.64, 1.12)	31 (2.7)	0.87 (0.59, 1.28)	0.80 (0.54, 1.21)
20–29	2,271 (56.0)	1.0	1.0	633 (55.1)	1.0	1.0
30–39	1,545 (38.1)	0.91 (0.83, 0.99)	0.96 (0.87, 1.06)	449 (39.1)	0.99 (0.87, 1.13)	1.03 (0.90, 1.19)
≥40	120 (3.0)	0.79 (0.62, 1.01)	0.86 (0.66, 1.12)	35 (3.1)	0.91 (0.63, 1.32)	0.97 (0.66, 1.42)
Education						
Primary	126 (3.1)	3.03 (2.22, 4.13)	1.65 (1.19, 2.30)	43 (3.8)	2.95 (2.04, 4.28)	1.66 (1.12, 2.47)
Secondary	2,729 (67.3)	2.04 (1.84, 2.26)	1.3 (1.16, 1.47)	829 (72.2)	2.10 (1.78, 2.49)	1.39 (1.16, 1.66)
Higher	809 (19.9)	1.0	1.0	187 (16.3)	1.0	1.0
Unknown	294 (9.7)	1.03 (0.88, 1.20)	0.94 (0.79, 1.12)	89 (7.8)	0.99 (0.76, 1.29)	1.02 (0.77, 1.36)
Marital status						
Married	2,141 (52.8)	1.0	1.0	598 (52.1)	1.0	1.0
Single	114 (2.8)	1.18 (0.91, 1.55)	1.01 (0.75, 1.24)	34 (3.0)	1.19 (0.82, 1.74)	1.01 (0.68, 1.50)
Unknown	1,803 (44.4)	1.29 (1.18, 1.42)	1.31 (1.18, 1.45)	516 (44.9)	1.20 (1.05, 1.36)	1.16 (1.01, 1.33)
BMI at first visit						
<18.5	191 (4.7)	0.85 (0.69, 1.03)	0.87 (0.70, 1.07)	44 (3.8)	0.79 (0.57, 1.09)	0.81 (1.01, 1.33)
18.5–24.9	2,047 (50.4)	1.0	1.0	533 (46.4)	1.0	1.0
25.0–29.9	1,080 (26.6)	1.13 (1.02, 1.26)	1.14 (1.02, 1.28)	323 (28.1)	1.26 (1.09, 1.47)	1.26 (1.08, 1.47)
≥30.0	727 (17.9)	1.31 (1.16, 1.49)	1.32 (1.15, 1.51)	246 (21.4)	1.59 (1.35, 1.88)	1.60 (1.34, 1.91)
Urban residency						
Urban	2,727 (67.2)	1.0	1.0	773 (67.3.)	1.0	1.0
Rural	1,331 (32.8)	1.06 (0.97, 1.17)	1.25 (1.13, 1.29)	375 (32.7)	1.03 (0.90, 1.18)	1.14 (0.99, 1.32)
Parity						
Primiparous	1,490 (36.7)	1.0	1.0	400 (34.8)	1.0	1.0
Multiparous	2,568 (63.3)	1.12 (1.03, 1.23)	1.0 (0.90, 1.11)	748 (65.2)	1.18 (1.03, 1.34)	1.01 (0.87, 1.17)
Test year						
2020	674 (16.6)	11.2 (8.77, 14.4)	10.1 (7.82, 13.02)	290 (25.3)	11.8 (7.58, 18.3)	10.2 (6.52, 15.98)
2021	2389 (58.7)	5.80 (4.71, 7.16)	5.2 (4.17, 6.45)	693 (60.4)	5.26 (3.44, 8.05)	4.49 (2.91, 6.94)
2022	884 (21.8)	2.08 (1.68, 2.58)	1.98 (4.17, 6.45)	142 (12.4)	1.36 (0.93, 2.29)	1.35 (0.86, 2.12)
2023	120 (2.9)	1.0	1.0	23 (2.0)	1.0	1.0

The adjusted models include the variables: BLL group (either above or below 5 or 10 µg/dL), maternal age, education, marital status, BMI, urban residency, parity and test year. BLL <5 µg/dL is used as a reference for the first model to compare it with ≥5 µg/dL group, and BLL <10 µg/dL is used as a reference for the second model to compare it with ≥10 µg/dL group.

## Discussion

This study assesses the predictors of lead exposure among pregnant women in the Adjara region of Georgia between the second half of 2020 and July 2023. While most pregnant women had BLL below 5 µg/dL (50.5%), a substantial proportion had elevated levels, with 35.5% having between 5-9.99 µg/dL and 14% having BLLs ≥10 µg/dL.

Our study showed that several sociodemographic factors are associated with elevated BLLs. Women with lower levels of education (primary or secondary) were significantly more likely to have elevated BLLs compared with those with higher education. Similar associations have been identified in a longitudinal study conducted in Mexico City by Tellez-Rojo, which found a relationship between lower levels of education and elevated BLLs during pregnancy.^12^ This finding suggests that education as a proxy for socioeconomic status is related to lead exposure. There are several possible explanations for this finding, including better housing, other economic means to buy consumer products, different dietary practices and different awareness of lead exposure risks and preventive measures. Other studies also support our findings, indicating that socioeconomic factors serve as predictors of elevated BLL.^13,14^

Additionally, geographic disparities in maternal BLL were observed, with higher relative odds of elevated BLLs in rural areas. It can be speculated that rural residents in Georgia could be exposed to lead via pathways that are not as relevant to urban settings, such as older housing with lead-containing paint, the lack of noncentralized water systems, or agricultural sources of contamination, including lead-containing pesticides, contaminated soil or locally produced spices, informal recycling, or traditional construction materials.^15^ These findings are in line with the study conducted in the state of Kansas that reported people in rural communities exhibited disproportionally higher risks of lead exposure compared to urban areas, where the risk of exposure was associated with factors such as lead-based paint, the persistence of noncentralized water systems, and industrial contamination sources.^16^ Further, studies have found that children in impoverished areas are more vulnerable to lead exposure due to factors like environmental contamination and inadequate housing.^17^

Maternal BMI emerged as a significant predictor of elevated BLLs. Women with BMI ≥30.0 had 60% higher odds of BLL ≥10 µg/dL compared with those with normal BMI (18.5–24.9). As seen in the investigation of Wells et al., maternal BMI was recognized as a relevant factor when evaluating lead exposure during pregnancy.^18^ This association may be attributed to the mobilization of lead stored in bone and adipose tissue during pregnancy, particularly in women with higher body fat.^19^

Notably, our study found no significant association of maternal age and parity with elevated BLLs, suggesting that the number of previous pregnancies does not substantially influence BLLs. It was similarly mentioned in the EDEN study in France that parity did not have an independent effect on the elevated BLLs.^20^

An important finding was the significant decline in elevated BLLs over the study period. Women tested in 2020 had 10 times higher odds of BLL ≥5 µg/dL compared with those tested in 2023, similar to that reported in a study conducted by Rylander et al.—the study examined trends in BLLs among pregnant and breastfeeding women in Adjara, Georgia. This temporal trend likely reflects the impact of public health interventions, such as the Adjara state health program for lead screening, awareness campaigns and national regulations that established thresholds for products that may contain lead, such as paint, spices, and toys.^21–24^ Several studies from Georgia have indicated that illegal adulteration of spices constitutes a significant source of lead exposure in the country, which led to a restriction on selling unpacked spices.^22^ These efforts originated with the MICS study, which uncovered alarming findings about BLL in children aged 2–7 years. The study results triggered a series of government initiatives that played a crucial role in reducing lead exposure among Georgian children. Additionally, until mid-2021, only women in high-risk groups were eligible for the lead biomonitoring program, which likely contributed to higher observed ORs; after mid-2021, testing became free and accessible for everyone. Since 2019, several studies from Georgia have shown that there has been a reduction in BLL across the country; for example, a study from Poti, Georgia, demonstrates significant progress in reducing lead exposure in children, likely attributable to recent public health initiatives.^25^ Another study, right after MICS, observed a notable decline in BLL among children within a relatively short timeframe, and this achievement can be credited to a combination of proactive measures implemented by public health authorities and heightened media attention across the country.^26^ Georgia launched a comprehensive State Health Program in May 2019, following the lead exposure findings of widespread elevated BLLs, with free follow-up testing, treatment and prevention measures for affected children and pregnant women.^7^ However, the persistence of elevated BLLs in 2023 indicates that further efforts are needed to achieve sustained reductions in lead exposure.^11^

The findings of this registry-based study provide significant insights into predictors of elevated BLLs among pregnant women in the Adjara region of Georgia between 2020 and 2023. Future research should assess why socioeconomic status, rural residency, and high BMI predict elevated BLLs in Adjara. Such studies could lead to the identification of important lead exposure sources or pathways. Despite global efforts to mitigate lead exposure, our results highlight the persistent burden of lead contamination in Adjara, with significant implications for maternal and fetal health.^27^

### Strengths and limitations

This study has several limitations and strengths. Only selected women were included in the first year, due to the program requirements, potentially introducing selection bias. Due to the varying selection criteria for pregnant women to undergo testing during the study period, we may have included women from high-risk groups in 2020, whereas from mid-2021 onward, testing became available to all women. The decision to perform the test depends on how the doctor explains it to the woman and her perception of the topic. However, given the wide range of risk factors that could qualify a woman for testing, we believe that the differences in selection criteria will not significantly affect our results. We did not have data on other factors affecting BLL during pregnancy, such as occupation, behavioral factors, smoking, and diet. The data only cover those who attended ANC and underwent BLL testing; therefore, data for some women are missing. In our study, we did not have a direct measure of deprivation or socioeconomic status due to the limitations of the available datasets. However, we used maternal education level as a proxy for socioeconomic status, as it is a well-established indicator of socioeconomic conditions in public health research. Additionally, rural residency was included as a variable, as it may reflect geographic disparities in access to resources and environmental risk factors, which are often linked to deprivation. Another limitation of the study is that it was conducted in Adjara, a region characterized by a diverse religious population and cultural practices, which hampers generalization to other regions. The strength of this study is based on two major national data sources – the lead biomonitoring program of pregnant and breastfeeding women and the GBR. These databases provide a robust sample size with high-quality standardized data and benefit from coordinated collaboration among the involved institutions.

## Conclusion

Low education level, high maternal BMI, rural residency, and test year were identified as significant predictors of BLL ≥5 µg/dL. These findings underscore the need for continued investment in lead poisoning prevention programs, particularly in emerging regions, to protect maternal and fetal health from the toxic effects of lead.
